# Short and long-term associations between serum proteins linked to cardiovascular disease and particle exposure among constructions workers

**DOI:** 10.5271/sjweh.4071

**Published:** 2023-02-27

**Authors:** Anda R Gliga, Karin Grahn, Per Gustavsson, Petter Ljungman, Maria Albin, Jenny Selander, Karin Broberg

**Affiliations:** 1 Institute of Environmental Medicine, Karolinska Institutet, Stockholm, Sweden; 2 Centre for Occupational and Environmental Medicine, Region Stockholm, Sweden; 3 Department of Cardiology, Danderyd Hospital, Stockholm, Sweden; 4 Division of Occupational and Environmental Medicine, Lund University, Sweden

**Keywords:** biomarker, cardiovascular biomarker, occupational exposure, respirable crystalline silica

## Abstract

**Objectives:**

Construction workers are exposed to respirable dust, including respirable crystalline silica (RCS), which is a potential risk factor for cardiovascular disease (CVD). The aim of this study was to evaluate whether exposure to particles among construction workers is associated with short- and long-term alterations in CVD-related serum proteins.

**Methods:**

Using proximity extension assay, we measured 92 serum proteins linked to CVD among active male construction workers (N=65, non-smokers) sampled on two occasions: during work and after vacation. First, we used linear models to identify short-term changes in proteins associated with particle exposure (assessed as respirable dust and RCS) during work. Secondly, we used linear mixed models to evaluate whether these associations were long-term, ie, persistent after vacation.

**Results:**

The median exposure to respirable dust and RCS during work were 0.25 mg/m^3^ and 0.01 mg/m^3^, respectively. Respirable dust was associated with short-term changes in six proteins (tissue factor, growth hormone, heme oxygenase-1, dickkopf-related protein-1, platelet-derived growth factor-B, stem cell factor); long-term associations were observed for the former three proteins. RCS was associated with short-term changes in five proteins (carcinoembryonic antigen-related cell adhesion molecule-8, hydroxyacid oxidase-1, tissue factor, carbonic anhydrase-5A, lectin-like oxidized LDL receptor-1); long-term associations were observed for the former four proteins.

**Conclusions:**

Moderate exposure to particles in the construction industry is associated with both short- and long-term changes in circulating CVD-related proteins. Further studies are needed to evaluate if these changes are predictors of occupationally induced clinical CVD.

Environmental exposure to particulate matter is a major contributor to increased morbidity and mortality related to cardiovascular disease (CVD) (1). Twelve key characteristics of cardiovascular toxicants have recently been put forward, and cardiovascular toxicity incurred by particular matter has been associated with seven key characteristics, eg, oxidative stress, inflammation, endothelial and vascular function, as well as hormone signaling (2).

Exposure to particles in occupational settings is in general higher compared with particle concentrations in the environment (3). In addition, the composition and the size of the particles is often different between the occupational and general environments. The construction industry is responsible for large amounts of particle emissions and construction workers have a high likelihood of particle exposure and thus subsequent risk of adverse effects. Of particular concern is occupational exposure to respirable crystalline silica (RCS), which is associated with silicosis and lung cancer and is classified as a group 1 carcinogen [The International Agency for Research on Cancer (IARC) 100C]. RCS exposure has also been identified as a cause of CVD [especially pulmonary heart disease (4–6)] and autoimmune rheumatic diseases (eg, systemic sclerosis and rheumatoid arthritis) (7), which in turn are associated with increased risk for CVD (8).

However, data identifying the effect levels of RCS is still scarce. A challenge is that exposure to RCS and exposure to respirable dust in occupational settings are interlinked, and it is therefore difficult to disentangle the effects of RCS from the effect of other mineral dusts. Further, the underlying CVD-triggering mechanisms of RCS and other particles in the construction industry are unclear, but the identification of associated biomarkers of effect can support mechanisms of action. A common drawback of many studies is the lack of smoking data, or the difficulty of adjusting for smoking (when this data exists), since smoking is an important risk factor for CVD. In general, it is not known whether the health effects of occupational exposure to particles and RCS are reversible upon exposure cessation, such as with vacation.

This is an exploratory study aimed at investigating whether low-to-moderate exposure to respirable dust and RCS among non-smoking construction workers leads to short-term (ie, associations present during work) as well as long-term changes (ie, associations that are persistent after vacation) in multiple serum proteins linked to CVD.

## Methods

### Study design

The study group consisted of construction workers (N=65) with exposure to particulate matter and RCS who were recruited 2018/2019 from 20 companies in the Stockholm County, as previously described (9). The inclusion criteria were that the participants should be males, non-smokers for the last six months and have worked in the construction industry for at least six months. At recruitment, the participants completed a questionnaire regarding age, country of birth, education, current residence, medical history, personal/family history of CVD, diet, physical activity, current as well as previous smoking history, use of snus (Swedish moist tobacco) and alcohol consumption. Additionally, the questionnaire covered former and current work including years in profession (years), exposure to noise, exposure to other particles during work (ie, diesel fumes, welding, dust other than RCS) or from hobbies. Biological samples were collected twice for each participant: during work (ie, in the morning before participants started the working day, and the same day as the exposure measurements) and after vacation (but before returning to work) as previously described (9). During the measurements after vacation, participants completed another short questionnaire about vacation activities involving exposure to RCS, health status, and tobacco use. Peripheral blood samples were collected in the same way at both timepoints in Becton Dickinson (BD) vacutainers for serum, allowed to clot at room temperature for 15 minutes and then centrifuged at 2500 rcf for 10 minutes. Upon separation, serum samples were aliquoted and kept on dry ice for transportation and then stored at −80 °C until analysis. Samples were shipped on dry ice to Clinical Biomarkers Facility, Scilife Lab, Uppsala University for protein analysis.

### Exposure assessment

RCS and respirable dust concentrations (filter measurements), were measured during one working day at each participant’s worksite as previously described (9). The filter for RCS and respirable dust was placed at the participant’s shoulder, close to the breathing zone. RCS and respirable dust were collected on 25 mm membrane filter in cassette holders with preseparator aluminium cyclone (SKC Inc, USA) using AirChek XR5000 sample pumps with a flow of 2.5 L/min. Airflow was measured using ChekMate flow meter model 375-07550 (SKC Inc, USA).

RCS and dust collected on membrane filters were analyzed at the accredited Analysis Laboratory, University Hospital, Örebro, Sweden, using gravimetric and x-ray diffraction analysis to determine the mass of respirable dust and silica, respectively, in mg (respirable dust: SS-EN 481 1993, respirable silica: SS-ISO 16258-2:2015 Arbetsplatsluft - Bestämning av kristallin kiseldioxid med röntgendiffraktion). The level of detection (LoD) for respirable silica was 0.002 mg/sample, and for respirable dust 0.10 mg/sample. Samples with levels below LoD were assigned the value 0 mg/m^3^ in the analyses. The results are presented as mg/m^3^ based on the sampled air volume. For every participant, we observed the usage and the duration of use of respirator masks the type (filter or air-supplied mask).

### Protein measurement

Proximity Extension Assay (PEA) technology (Olink Proteomics, Uppsala, Sweden) was used to measure 92 unique serum proteins related to cardiovascular processes (cardiovascular panel II). The proteins from the panel are connected to the following gene ontology terms: angiogenesis (N=16), blood vessel morphogenesis (N=16), catabolic process (N=20), cell adhesion (N=29), coagulation (N=10), heart development (N=5), immune response (N=38), inflammatory response (N=28), MAPK cascade (N=25), platelet activation (N=9), proteolysis (N=16), regulation of blood pressure (N=8), response to hypoxia (N=8), response to peptide hormone (N=11), wound healing (N=14), other gene ontology terms (N=10). In addition, the proteins are associated with the following diseases: cardiovascular (N=58), cancer (N=66), cutaneous (N=19), digestive (N=24), hemic (blood) and lymphatic (N=24), hepatic (N=15), infectious (N=23), inflammatory (N=32), metabolic (N=36), neurological (N=47), pulmonary (N=31), renal (N=28), skeletal (N=23), other (N=58). The analyzed proteins are included in supplementary material, www.sjweh.fi/article/4071, table S1.

One μL of serum was used for the analysis. Processing, quality control as well as normalization were performed as previously described (9). Protein levels were reported as normalized protein expression (NPX) values on a log2-scale. The proteins ITGB1BP2 and BNP were removed from the analyses due to >60% samples under LoD; the statistical analyses were performed on 90 proteins.

### Statistics

*Data exploration using principal component analysis (PCA)*. PCA heatmaps were constructed using the *prince.plot* function in the swamp package in R for the sampling timepoints during work and after holidays. The function reduces the dimensions of the protein data to so called principal components that explain part of the variation in the protein data set and then tests each variable (supplementary figure S1) against these components to evaluate possible associations. The variables that were associated with the principal components was considered as possible covariates when constructing the linear models. Heatmaps depict p values (− log10-transformed) of these associations. Hierarchical clustering of the variables was generated using the *hclust* function.

*Evaluation of differentially expressed proteins*. For the cross-sectional analysis during work, we used linear models adjusted for body mass index (BMI) to evaluate associations between exposure to respirable dust or RCS and serum proteins at timepoint 1 (during work). The selection of the covariates (ie, BMI) was based on the PCA heatmap.

For the longitudinal analysis, we used linear mixed models to evaluate if there is a reversible or persistent association between exposure to respirable dust or RCS, and serum proteins, despite exposure cessation during vacation. We used protein data from the two timepoints (during work and after vacation) as input. The analysis was done using the *lmer* function in the *lme4* package in R. The mixed models included participants as random factors (random intercepts) and BMI as well as respirable dust or RCS as fixed factors. Variance explained by fixed factors (R^2^m) was calculated using *RsqGLM* function from the R package *MuMin*. The models were not adjusted for other factors than BMI since they were not associated with protein variation, as indicated by the PCA heatmap.

All analyses ware performed using R v.4.0.4.

## Results

### Characteristics of the study participants

All study participants were males working within the construction industry. The participants had a relatively healthy lifestyle: they were physically active, non-smokers [with the exception of four individuals who were occasional (party) smokers], had low alcohol consumption and a relatively high intake of vegetables (table 1). The median exposure to respirable dust and RCS was 0.25 mg/m^3^ (min–max: 0–2.06) and 0.01 mg/m^3^ (min–max: 0–0.21), respectively. None of the workers had levels of exposure to respirable dust that were higher than the Swedish occupational exposure limit (OEL, 2.5 mg/m^3^), but three individuals were exposed to RCS at levels above the European and the Swedish OEL (0.1 mg/m^3^). There was a high correlation (r_S_ = 0.82) between respirable dust and RCS. On average, 4% of the respirable dust consisted of RCS.

**Table 1:** Characteristics of the study group of construction workers.

	N	Median (min–max)	N (%)
Continuous variables			
Age (years)	65	38 (20–65)	
Body mass index (kg/m^2^)	65	26.7 (19.3–45.9)	
Years working in construction work sector	65	10 (0–43)	
Respirable dust (<4 µm, mg/m^3^) ^a^	65	0.25 (0–2.06)	
Respirable crystalline silica (mg/m^3^) ^a^	65	0.01 (0–0.21)	
Vacation before second sampling (weeks)	60	4 (0–10)	
Categorical variables ^b^			
Country of birth (Sweden)	65		45 (69)
Higher education (university)	65		5 (8)
Smoking status (current)	65		4 (6) ^c^
Smoking status (ever)	63		26 (41)
Current snus use	65		24 (37)
Alcohol consumption (≥4 times/week)	64		12 (19)
Vegetable consumption (≥5 times/week)	64		44 (69)
Intake of fish (≥ once/week)	64		31 (48)
History of cardiovascular disease	65		17 (26)
History of lung disease	65		23 (35)
Physical activity (high) ^d^	64		30 (47)

a Evaluated using personal sampling of particles on filters.

b Percent calculated from complete answers.

c All reported that they were party smokers.

d Once a week or more of ≥30 minutes of regular physical activity.

We performed PCA analysis to evaluate to what extent the characteristics of the study participants explained the variation in levels of the serum proteins. After hierarchical clustering, BMI was the top variable that was associated with variation of the protein levels (supplementary figure S1). The association with BMI was more significant after vacation (supplementary figure S1B) compared with during work (supplementary figure S1A).

### Differentially expressed proteins in relation to respirable dust exposure

We observed in cross-sectional linear models that exposure to respirable dust during work (timepoint 1) was negatively associated with six proteins (TF, tissue factor; GH, growth hormone; HO-1, heme oxygenase 1; DKK1, Dickkopf-related protein 1; PDGFsubunit B, platelet-derived growth factor subunit B; SCF, stem cell factor) (table 2).

**Table 2:** Differentially expressed proteins in serum in relationship to exposure to respirable dust analyzed using linear models (during work, cross-sectional analysis, N=64) and linear mixed-effects models (during work and after vacation, longitudinal analysis, N=125). The proteins significant in both analyses are marked in bold. None of the associations between respirable dust exposure and measured proteins remained statistically significant after adjustment for multiple testing. (Bonferroni threshold, 0.05/92=5.4 *10-4) [SE=standard error.]

Protein	Cross-sectional – during work (linear models, N=64)			Longitudinal – during work and after vacation (linear mixed-effects models, N=125)		
	
	R^2^(%) ^a^	Beta (SE) ^b^	P-value ^c^	R^2^_m_ (%) ^d^	Beta (SE) ^e^	P-value ^f^
**TF**	**10**	**-0.25 (0.08)**	**0.004**	**9**	**-0.21 (0.07)**	**0.005**
**GH**	**14**	**-1.39 (0.57)**	**0.018**	**12**	**-1.1 (0.51)**	**0.031**
**HO-1**	**8**	**-0.28 (0.12)**	**0.026**	**12**	**-0.24 (0.10)**	**0.013**
DKK1	4	-0.28 (0.13)	0.031	4	-0.18 (0.10)	0.070
PDGF subunit B	4	-0.27 (0.13)	0.038	4	-0.17 (0.09)	0.063
SCF	24	-0.22 (0.11)	0.043	21	-0.17 (0.10)	0.073
HAOX1	17	0.64 (0.43)	0.144	20	0.88 (0.35)	0.013
IL1RL2	2	0.15 (0.12)	0.202	10	0.25 (0.10)	0.014
AMBP	2	-0.1 (0.06)	0.090	6	-0.1 (0.05)	0.040
GT	0	0.2 (0.15)	0.165	5	0.27 (0.13)	0.041
SPON2	1	-0.08 (0.06)	0.183	7	-0.09 (0.04)	0.047

a Variance in proteins levels explained by the linear model.

b Regression coefficient from linear models interpreted as standard deviation difference in protein levels per unit respirable dust increase adjusted for body-mass index.

c P-value from the linear model to test the association between protein in serum and respirable dust as exposure variable.

d Variance explained by fixed factors (respirable dust, body-mass index).

e Regression coefficient from linear mixed models interpreted as standard deviation difference in protein levels per respirable dust unit increase, adjusted for body- mass index as fixed factors, and participant as random factors.

f P-value from test of contribution of respirable dust to protein variance using an analysis of variance approach with Satterthwaite approximation for degrees of freedom.

**Figure 1:**
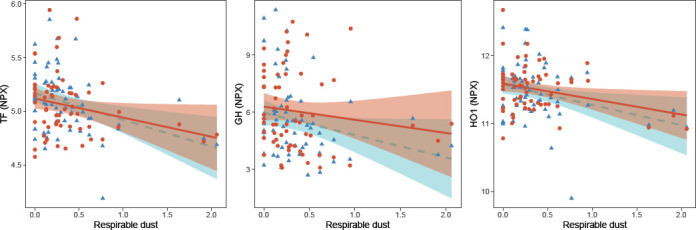
Scatter plots of the serum levels of TF, GH and HO1 (normalized protein expression, NPX) and exposure to respirable dust (mg/m3), during work (blue triangles) and after vacation (red dots). Lines represent the regression lines (dashed blue line during work, and solid red line after vacation) and the shadows depict the 95% confidence interval.

Next, we evaluated by linear mixed-effect models whether these changes were long-term, ie, persistent after vacation (table 2). Respirable dust was associated with three of the six proteins in the longitudinal analysis, namely TF, GH and HO-1, while there was a recovery effect for the other three proteins, DKK1, PDGFsubunit b and SCF) (table 2, figure 1). The direction of association and the effect estimates for TF, GH and HO-1 were similar between the cross-sectional and longitudinal analyses. The variance in protein levels explained by the linear model as well as the variance explained by the fixed factors (BMI, respirable dust) in the linear mixed models was approximately 10% for these three proteins (table 2). In addition, we identified five proteins (HAOX1, hydroxyacid oxidase 1; IL1RL2, interleukin 1 receptor like 2; AMBP, alpha-1-microglobulin/bikunin precursor; GT, gastrotropin; SPON2, spondin2) that were significant in the longitudinal analysis, but that were not identified in the cross-sectional analysis (table 2). None of the associations between respirable dust exposure and measured proteins remained statistically significant after adjustment for multiple testing.

### Differentially expressed proteins in relation to RCS

**Figure 2:**
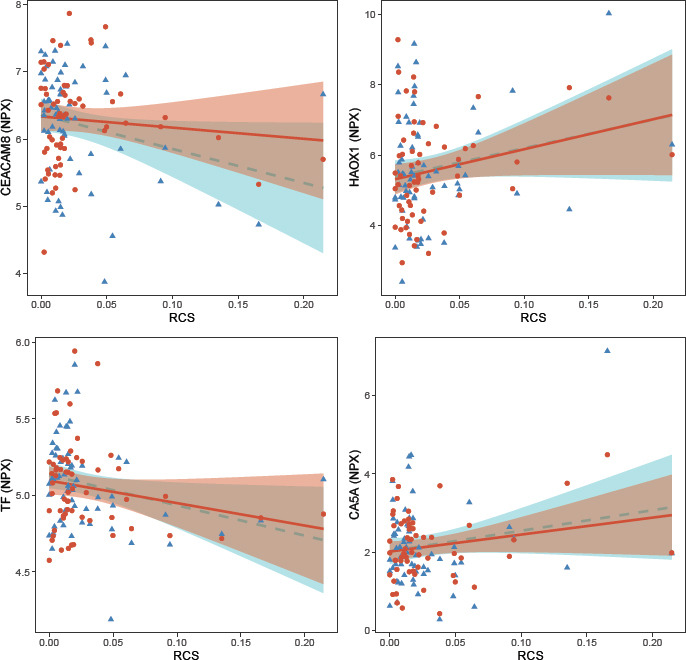
Scatter plots of the serum levels of CEACAM8, HAOX1, TF and CA5A (normalized protein expression, NPX) and exposure to respirable crystal[1] line silica (RCS), during work (blue triangles) and after vacation (red dots). Lines represent the regression lines (dashed blue line during work, and solid red line after vacation) and the shadows depict the 95% confidence interval.

Using a similar approach for RCS as for the exposure to respirable dust, we first performed a cross-sectional analysis and observed associations (P<0.05) between RCS during work (timepoint 1) and five proteins: LOX1, lectin-like oxidized LDL receptor 1; CEACAM8, carcinoembryonic antigen-related cell adhesion molecule 8; HAOX1, hydroxyacid oxidase 1; TF; and CA5A, carbonic anhydrase 5A, mitochondrial (table 3). RCS was positively associated with three proteins (LOX-1, CEACAM8, TF) and negatively associated with two proteins (HAOX1, CA5A). In the longitudinal analyses, we found that RCS was associated with four of the five proteins (CEACAM8, HAOX1, TF, CA5A) (table 3, figure 2). The direction of association for these four proteins were similar between the cross-sectional and longitudinal analyses. The variance in protein levels explained by the linear model and the variance explained by the fixed factors (BMI, RCS) in the linear mixed models for these proteins varied between approximately 5–25% (table 3). In addition, we identified one protein (PAR-1, proteinase-activated receptor 1) that was significant in the longitudinal analysis but not in the cross-sectional analysis (table 3). None of the associations between RCS exposure and measured proteins remained statistically significant after adjustment for multiple testing.

**Table 3:** Differentially expressed proteins in serum in relationship to exposure to respirable crystalline silica analyzed using linear models (during work, cross-sectional analysis, N=64) and linear mixed-effects models (during work and after vacation, longitudinal analysis, N=125). The proteins significant in both analyses are marked in bold. None of the associations between RCS exposure and measured proteins remained statistically significant after adjustment for multiple testing. (Bonferroni threshold, 0.05/92=5.4 *10-4) [SE=standard error.]

Protein	Cross-sectional – during work (linear models, N=64)			Longitudinal – during work and after vacation (linear mixed-effects models, N=125)		
	
	R^2^(%) ^a^	Beta (SE) ^b^	P ^c^	R^2^m (%) ^d^	Beta (SE) ^e^	P ^f^
LOX-1	13	-5.06 (2.04)	0.016	9	-2.89 (1.48)	0.052
**CEACAM8**	**8**	**-5.46 (2.50)**	**0.033**	**6**	**-3.67 (1.81)**	**0.043**
**HAOX1**	**20**	**9.64 (4.47)**	**0.035**	**20**	**9.71 (3.74)**	**0.009**
**TF**	**4**	**-1.97 (0.92)**	**0.036**	**6**	**-1.68 (0.81)**	**0.038**
**CA5A**	**21**	**6.36 (3.16)**	**0.049**	**25**	**5.88 (2.38)**	**0.013**
PAR-1	2	-1.6 (0.88)	0.073	3	-1.78 (0.90)	0.047

a Variance in proteins levels explained by the linear model.

b Regression coefficient from linear models interpreted as standard deviation difference in protein levels per unit respirable crystalline silica increase, adjusted for body-mass index.

c P-value from the linear model to test the association between protein in serum and respirable crystalline silica as exposure variable.

d Variance explained by fixed factors (respirable crystalline silica, body-mass index).

e Regression coefficient from linear mixed models interpreted as standard deviation difference in protein levels per respirable crystalline silica increase, adjusted for body-mass index as fixed factors, and participant as random factors.

f P-value from test of contribution of respirable crystalline silica to protein variance using an analysis of variance approach with Satterthwaite approximation for degrees of freedom.

## Discussion

In this study, we found that exposure to respirable dust and RCS during work was associated with short-term changes in CVD-related proteins, which is indicative of acute effects. In addition, particle exposure was also associated with long-term changes in some of those proteins, which is suggestive of chronic effects. It is possible that some of the short-term changes are a result of chronic exposure to respirable dust and RCS, but which resolve after holidays. Importantly, these associations were observed at levels of exposure below the OEL for respirable dust and RCS. The long-term associations could potentially be related to the long persistence of particles in the respiratory tract, which is of particular concern for RCS. For example, a recent *in vivo* study indicated that single intratracheal instillation of quartz in rats resulted in long-term persistence of particles inside the lungs, chronic inflammation and neoplastic lesions after 96 weeks (10). Despite the high correlation between respirable dust and RCS (r_s_= 0.82) only one protein (TF) was associated with both exposures during work, and there was no recovery after vacation. This could indicate that the associations with respirable dust and RCS are mediated to some extent by different pathways.

We tested 92 proteins that are part of the CVD II panel by Olink. The selection of the proteins was based on the gene ontology terms and diseases related to cardiovascular processes but not exclusively; a large majority of the proteins are related to CVD (58/92) and cancer (66/92). The CVD-related proteins are part of processes related to particle-induced CVD and key characteristics of cardiovascular toxicants, such as inflammatory response, blood coagulation and regulation of blood pressure (supplementary table S2) (2, 11). Low-grade systemic chronic inflammation is a mechanism of particle toxicity that contributes to CVD (2) and several of the tested proteins (28 out of 92) are associated with inflammation. However, low-grade inflammation is difficult to detect using canonical traditional biomarkers of inflammation and new reliable biomarkers of chronic inflammations have yet to be established (12). A multi-dimensional exploratory approach as used in this study is more likely to identify changes that are of relevance for particle exposure and inflammation/CVD.

The connection with cancer should be interpreted with caution since cancer is the most widely studied topic in biomedical sciences and many genes and proteins have been studied in the context of cancer (13). Nevertheless, in the context of occupational exposure to respirable dust and RCS, cancer is a relevant disease outcome as crystalline silica is classified as a group 1 carcinogen (IARC 100C).

### Short-term changes in serum proteins and exposure to construction particles

We identified four proteins that were associated with exposure to respirable dust (DKK1, PDGFb, SCF) or RCS (LOX1) during work, and for which the association was not present after vacation. This short-term association is suggestive of acute effects of exposure to particles that are seemingly resolved when exposure is interrupted. Each protein is discussed individually below (with references), but when relating our data to the scientific literature, the *lower* level of SCF could potentially be associated with an increased (albeit temporary) risk of CVD, while the *lower levels of* LOX-1 are suggestive of protection against CVD. As regards the other proteins the direction of association and toxic outcomes is unclear.

Stem cell factor (SCF) exists both as membrane-bound and soluble forms and is involved in hematopoiesis and vasculogenesis with importance in preserving the integrity of the cardiovascular system (14). In a prospective study, high levels of plasma SCF were associated with lower risk of cardiovascular events and death (15). In our study we found lower levels of SCF with increasing respirable dust during work, and with recovery after vacation, which may suggest a higher risk of CVD.

Lectin-like oxidized LDL receptor 1 (LOX-1) is a receptor for the oxLDL in endothelial cells and is involved in progression of atherosclerosis and tumorigenesis (16). LOX-1 serum levels are positively associated with risk of CVD such as acute coronary syndrome, stroke, and atherosclerotic plaque instability (17). LOX-1 is considered a possible biomarker for plaque stability and vulnerability in patients with coronary artery disease (18). An *in vivo* study in rats exposed to ambient particular matter reported an increased gene expression of *OLR1* (official name for gene coding for LOX-1) in the lung tissue together with other markers of inflammation, such as *HMOX1* (official name for gene coding for HO-1) (19). In addition, *in vivo* exposure to silica in rats resulted in an increased *OLR1* gene expression in lung tissue (20), while in our study we found a negative association with RCS (but not with respirable dust).

The Dickkopf1 (DKK1) protein is involved in the inhibition of the Wnt signaling pathway that has a role in cancer development and progression (21). DKK1 is overexpressed in various cancers (including lung squamous cell carcinoma) and is a putative biomarker for targeted therapy and a predictor for prognosis (21). Exposure to atmospheric PM2.5 (22) and to organic compounds from gasoline and biogasoline fuel emissions (15) in human lung cells (BEAS-2B) resulted in decreased gene expression of *DKK1*. In our study, serum levels of DKK1 were relatively lower in relation to respirable dust concentrations during work.

PDGF subunit B is a protein involved in vascular development and function (23). In patients with acute coronary syndrome, serum PDGF was increased, especially in the local coronary artery (24). In our study, serum levels of PDGF subunit B were relatively lower in relation to respirable dust during work.

### Long-term changes in serum proteins and exposure to construction particles

We identified six proteins that were associated with exposure to respirable dust (TF, GH, HO-1) or RCS (CEACAM8, HAOX1, TF, CA5A), and for which the association was maintained after vacation. However, it is at present unclear whether these changes constitute an adaptive response to chronic exposure to particles or predict CVD. When summarizing the direction of associations in our study in relation to the published literature, the lower long-term level of TF may suggest an adaptive response, whereas the lower long-term level of HO-1 an increased risk for CVD. For the rest of the proteins it was unclear whether the direction of association is related to adaptive or linked to an increased risk for disease.

Tissue factor (TF) is the initiator of the extrinsic blood coagulation cascade in response to tissue damage and is involved in the modulation of the inflammatory response of the blood vessels (25). TF is also highly expressed in cancer cells and can be shed into the systemic circulation, which can contribute to the increased risk of thrombosis in cancer, and it also promotes for cancer progression (26). A study showed that plasma TF was upregulated in lung cancer patients compared to the control (27). There is also evidence that the impairment of the cardiovascular system may involve upregulation of TF as an underlying mechanism. For example, *TF* gene expression (as well as expression of other genes related to TF signaling) was upregulated after exposure of human pulmonary artery endothelial cells to ultrafine fine air pollution particles (28). Additionally, exposure to PM10 induced *TF* gene expression in human differentiation macrophages and increased TF circulating levels after intratracheal instillation in rats (29). Another study reported increase in TF and TF-dependent coagulation after repeated-dose intratracheal instillation of PM2.5 in rats over one month (30). In our study, RCS and respirable dust exposures were associated with lower serum levels of TF and maintained after vacation, which may relate to an adaptive response of particle and RCS exposure.

Growth hormone (GH) is involved in several functions such as bone development in children, metabolism and heart development; both GH excess and deficiency in adults have been associated with higher morbidity and mortality related to CVD (31). To our knowledge, GH expression has not been associated with exposure to particular matter or RCS. In our study, respirable dust (but not RCS) was negatively associated with serum levels of GH and the association was maintained after vacation.

Heme oxygenase (HO-1) is an antioxidant enzyme that catalyzes degradation of heme to biliverdin, ferrous ion, and carbon monoxide. HO-1 has antioxidant, anti-inflammatory, and anti-apoptotic effects and plays an important role in maintaining antioxidant/oxidant homeostasis and in the prevention of vascular injury (32). *In vivo* and clinical studies indicate that HO-1 plays a protective role against the progression of atherosclerotic diseases (33). Exposure to standard quartz (DQ12) induced increased gene expression of *HMOX1* in rat alveolar macrophages (34) as well as in rat lung epithelial cells (35). Exposure to DQ12 also induced increased gene expression of *HMOX-1* in rat lung tissue after instillation (35). Similarly, there was upregulation of *HMOX-1* expression up to 16 weeks after inhalation of crystalline silica in rats (28). In addition, *in vivo* studies indicate that acute (but not subchronic) exposure to particulate matter increases gene expression of antioxidant genes, such as *HMOX-1*, in the lung and aortic tissues of rats (36). In our study, respirable dust (but not RCS) was associated with lower serum levels of HO-1 over time which may indicate less antioxidant defense and in turn higher CVD risk.

The human aldehyde oxidase (HAOX1) is a molybdenum enzyme involved in metabolism of xenobiotics (37). HAOX1 is part of phase I liver metabolism and has been shown to affect clinical efficiency of novel drugs and contribute to drug failure (38). In vitro exposure of lung cells to particles (gasoline combustion particles) resulted in decreased gene expression of *AOX1* gene (15). In our study, exposure to RCS during work was positively associated with serum levels of HAOX1 and this association was persistent after vacation.

Carbonic anhydrase 5A (CA5A) is part of a family of enzymes that catalyze the CO_2_ hydration and are involved in maintenance of pH and metabolic rate (39). To our knowledge CA5A expression has not been associated with neither CVD, cancer, exposure to particular matter nor RCS.

Carcinoembryonic antigenrelated cell adhesion molecule 8 (CEACAM8) belongs to the carcinoembryonic antigen (CEA) family which includes molecules involved in cell adhesion and migration, as well as pathogen binding (40). Ceacam8 is exclusively expressed in granulocytes and constitutes a marker of granulocyte activation. For example, CEACAM8 is involved in regulating adhesion, superoxide production and degranulation of eosinophils (41). CEACAM8 is overexpressed in cancer tissue, in particular lung and colorectal cancer (proteinatlas.org). To our knowledge, CEACAM8 expression has not been associated with exposure to particular matter or RCS. In our study, exposure to RCS during work was negatively associated with serum levels of CEACAM8 and this association was persistent after vacation.

### Strengths and limitations

One limitation of the study is the relatively low sample size, that can result in a low statistical power and explain the lack of significant findings when adjusting for multiple testing. The adjustment for multiple testing is performed on the assumption that the tested proteins are independent of each other. However, many proteins belong to the same GO category and are part of similar biological pathways. So, from a biological perspective these proteins do not necessarily act independent of each other. Therefore, the multiple testing adjustment in this case could lead to an over adjustment. In addition to looking only at the P-value we should also consider the effect estimate which is likely more biologically relevant. Another limitation is that we did not evaluate protein levels in unexposed individuals, but by repeated sampling we could distinguish between short- and long-term effects among exposed workers. Another limitation is the lack of adjustment for personal protection equipment (such as respiratory protection). However, the use of such protection was in general low (17% of individuals) and under very short intervals of time, therefore we do not believe it considerably influenced the overall exposure to respirable dust and RCS. However, personal protection, if used correctly, probably have influenced the peak exposure. Additionally, the exposure measurement was only performed on one occasion during work, and the exposure to respirable dust and RCS likely varies over time. However, most of the participants were working at the same working site for several weeks, and it seems plausible that the exposure was in the same level during the weeks prior to the exposure measurements. Further, in order to limit potential diurnal fluctuations in the protein levels, the blood samples were taken at the same time during the day at both time points. Finally, the linear models were constructed using BMI as a covariate as indicated by the PCA heatmap, however this may be an over adjustment, if BMI is part of the causal chain for the observed changes in protein levels.

Some of the strengths of the study constitute the inclusion of only non-smoking men (though four individuals identified themselves as party smokers in the questionnaire), which reduces the potential confounding of smoking. In addition, we had access to information on lifestyle factors that can influence the risk of CVD and that were considered in the PCA prior to the construction of the statistical models.

### Concluding remarks

Our study indicates that occupational exposure to particles in the form of respirable dust and RCS at levels within the OEL was associated with changes in serum proteins with putative cardiovascular function and that are also partly involved in pathways that define the key characteristics of cardiovascular toxicants (2). It is unclear if the protein changes observed in this study are early biomarkers predictive of CVD or an adaptive response to particle exposure in occupational settings. For some proteins the direction of association was indicative of adaptive response or protection against CVD (eg, TF), while for some other proteins the direction of association was suggestive of increased risk of CVD (eg, HO-1). From a biological perspective, this is to be expected since a high level of regulation is taking place with the goal to maintain a functional balance. Finally, we emphasize that this is an exploratory study and the findings should be confirmed in future studies.

### Funding

AFA Insurance in Sweden (Grant number 160361) and the Karolinska Institutet funded this study.

## Supplementary material

Supplementary material
