# Supplementary material for: Short and long-term associations between serum proteins linked to cardiovascular disease and particle exposure among constructions workers
Source: Scand J Work Environ Health. 2023 Feb 27;49(2):145–54. doi: 10.5271/sjweh.4071 (PMC10577013; doi:10.5271/sjweh.4071)
Supplement: Supplementary material [file SJWEH-49-145-S001.pdf]

Short and long-term associations between serum proteins linked to cardiovascular disease and particle exposure among constructions workers[1]

by Anda R Gliga, Karin Grahn, Per Gustavsson, Petter Ljungman, Maria Albin, Jenny Selander, Karin Broberg [2]

1. Supplementary material
2. Correspondence to: Karin Broberg, Institute of Environmental Medicine, Karolinska Institutet, Box 210, 171 77 Stockholm, Sweden. [E-mail: karin.broberg@ki.se]

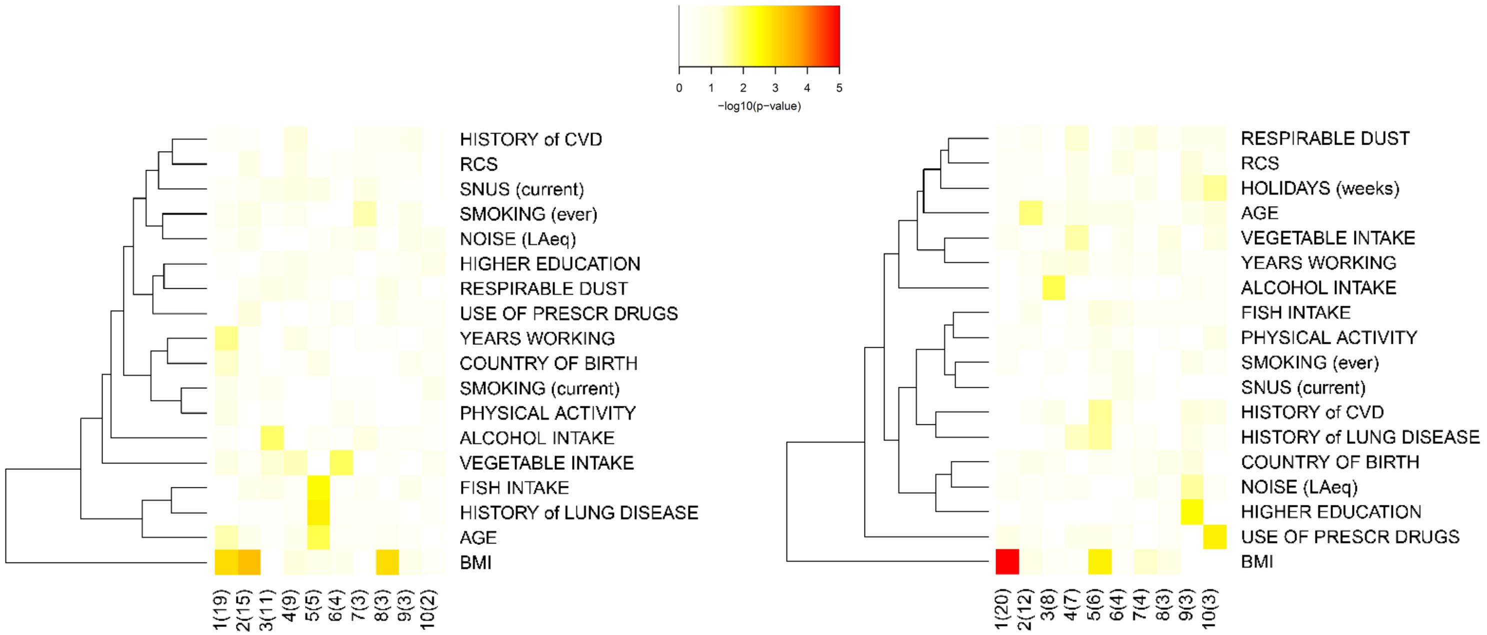

Supplementary figure 1. Heatmaps of the principal components (PC) that explain the protein serum variation in the study group during work (left) and after vacation (right). Numbers on the horizontal axis of the heatmaps indicate number of the principal component and in brackets is the percentage of the explained variation in the protein dataset. Heatmaps were constructed using input data from linear regression of association between the principal components of the protein data and the selected variables. The influence of the variables on the overall variation of the protein dataset is plotted in a heatmap based on the p-value of the association. Input data was the normalized protein expression values (on a log2-scale). “country of birth” is categorized as Sweden or outside Sweden; “Vegetable intake”, “Fish intake”, “Alcohol intake”, “Physical activity” are categorized as high or low; “History of CVD”, “History of lung disease”, “Snus (current)”,” Smoking (ever)”, “Smoking (current)”, are categorized as “yes” and “no”; RCS (respirable crystalline silica), and respirable dust are measures of exposure to particles and are continuous variables; LAeq, Equivalent continuous sound level (decibels). Smoking (current) refers to party smoking.

Supplementary table S1. Normalized protein expression values during work and after vacation for the 92 analyzed proteins. Data presented as median, min and max.

	Timepoint 1 - during work			Timepoint 2 - after vacation		
**Protein symbol**	**Median**	**Min**	**Max**	**Median**	**Min**	**Max**
ACE2	3,81	2,98	6,35	3,89	2,93	6,76
ADAMTS13	7,27	7,08	7,54	7,28	6,14	7,56
ADM	5,71	3,39	6,69	5,89	4,93	6,81
AGRP	4,28	1,9	6,84	4,25	3,21	6,28
AMBP	7,55	6,57	7,87	7,55	7,21	7,99
ANGPT1	10,71	9,56	11,49	10,92	10,07	11,48
BMP6	4,25	2,78	4,96	4,37	2,48	5,5
BNP	0,53	0,16	2,15	0,63	0,1	3,2
BOC	4,22	3,04	4,81	4,1	3,42	4,98
CA5A	1,98	0,29	7,13	1,99	0,44	4,49
CCL17	9,01	6,94	11,55	9,46	7,66	11,37
CCL3	6,89	5,25	7,92	6,87	5,68	8,47
CD4	4,89	3,4	5,72	4,91	4,32	5,43
CD40L	7,37	4,92	8,76	7,41	5,01	9,1
CD84	5,48	3,74	6,04	5,56	3,79	6,19
CEACAM8	6,38	3,88	7,41	6,34	4,32	7,87
CTRC	10,18	8,42	11,99	10,09	8,54	11,62
CTSL1	6,45	5,17	7,39	6,54	5,82	8,17
CXCL1	9,39	6,5	10,36	9,72	8,6	10,62
DCN	4,87	3,68	5,31	4,92	4,53	5,38
DECR1	3,86	2,25	8,76	3,66	2,25	5,6
DKK1	10,5	8,48	11,4	10,56	9,47	11,36
FABP2	8,4	6,04	9,8	8,73	7,03	10,56
FGF21	5,8	2,95	8,82	6,33	2,54	10,05
FGF23	2,44	1,56	3,27	2,39	1,51	4,31
FS	11,04	8,93	12,32	11,05	10,23	11,95
GAL9	7,57	6,98	8,68	7,61	6,88	8,42
GDF2	9,02	6,44	9,72	9,03	7,66	10,06
GH	5,2	2,7	11,37	5,46	3,08	10,75
GIF	7,68	6,29	9,06	7,59	6,31	9,12
GLO1	6,46	4,65	8,21	6,45	5,76	7,51
GT	1,6	0,75	2,71	1,55	0,84	3,58
HAOX1	5,42	2,41	10,02	5,41	2,95	9,28
HBEGF	5,88	4,25	7,34	6,05	5,29	7,06
HO-1	11,49	9,9	12,41	11,55	10,78	12,67
HOSCAR	10,56	8,98	11,12	10,61	9,93	10,97
HSP27	7,53	4,7	10,06	7,54	6,5	9,37
IDUA	5,76	4,65	6,67	5,83	5,16	6,75
IgG-FcR-IIb	3,37	1,93	4,98	3,34	2,1	4,74
IL16	6,38	3,73	9,53	6,36	5,48	7,7
IL17D	1,85	1,02	2,52	1,83	0,76	2,72
IL18	8,47	7,07	9,55	8,51	7,7	9,55

IL1RA	4,64	3,41	6,61	4,72	3,96	5,79
IL1RL2	4,49	3,64	5,34	4,46	3,28	5,29
IL27	5,31	3,97	6,23	5,26	4,35	6,12
IL4RA	1,86	1,14	4,13	1,95	1,09	4,78
IL6	2,14	1,11	5,23	2,4	1,42	5,94
ITGB1BP2	1,29	0,68	3,78	1,38	0,58	2,93
KIM1	6,16	4,52	8,26	6,26	4,57	8,06
LEP	5,69	2,68	8	6,11	3,05	8,23
LOX1	9,12	7,23	10,09	9,19	6,99	10,14
LPL	9,14	8,1	10,02	9,15	7,91	9,97
MARCO	6,49	5,98	6,89	6,54	6,2	7,12
MERTK	5,81	4,02	6,6	5,86	5,38	6,73
MMP12	6,42	4,8	8,26	6,62	5,15	8,42
MMP7	12,1	10,44	13,28	12,1	10,5	13,18
NEMO	4,04	2,23	6,83	4,03	3,26	5,27
PAPPA	2,94	1,33	4,38	2,96	1,74	4,46
PAR1	5,12	3,86	5,86	5,1	4,69	8,3
PARP1	1,32	0,21	8,26	1,23	0,44	3,4
PDGF subunit B	11,01	8,93	11,47	11,19	9,92	11,55
PDL2	3,02	1,6	4,04	2,99	2,25	4,07
PGF	7,62	5,83	8,45	7,62	7,04	8,5
PIGR	6,55	6,14	6,79	6,5	6,28	6,78
PRELP	8,13	7,21	8,49	8,15	7,6	8,47
PRSS27	9,18	7,31	10,08	9,27	8,52	10,28
PRSS8	8,47	6,98	9,23	8,56	7,87	9,35
PSGL1	3,91	3,09	5,01	3,87	3,23	4,37
PTX3	4,17	2,77	5	4,11	3,29	5,06
RAGE	13,23	11,49	14,14	13,28	12,41	14,15
REN	6,51	5,14	7,67	6,51	5,26	7,84
SCF	9,18	7,8	9,63	9,14	7,63	9,73
SERPINA12	1,92	0,05	6,86	1,98	0,25	6,9
SLAMF7	3,9	2,13	6,12	3,93	2,07	6,19
SOD2	9,82	9,56	10,06	9,82	9,63	10
SORT1	9,1	7,69	9,5	9,13	8,76	9,74
SPON2	8,4	7,21	8,8	8,44	8,13	8,8
SRC	3,76	2,56	5,98	4,06	2,6	5,89
STK4	1,44	0,28	5,15	1,58	0,45	3,3
TF	5,07	4,19	5,85	5,02	4,58	5,94
TGM2	7,37	4,95	9,3	7,51	6,31	8,78
THBS2	5,68	4,78	6,24	5,64	5,13	6,17
THPO	3,38	1,76	4,17	3,46	2,82	4,11
TIE2	7,33	5,83	7,87	7,35	6,83	7,77
TM	10,54	8,09	11,1	10,53	9,75	11,23
TNFRSF10A	3,17	2,01	4,45	3,22	2,46	4,37
TNFRSF11A	5,6	3,87	6,33	5,67	4,61	6,41
TNFRSF13B	9,6	8,19	10,76	9,6	8,43	10,65

TRAILR2	5,32	3,78	6,05	5,36	4,65	6,16
VEGFD	7,48	3,81	8,27	7,5	3,87	8,25
VSIG2	4,2	2,61	5,31	4,28	3,37	5,54
XCL1	4,72	2,87	7,91	4,75	3,67	7,89

Supplementary table S2. Overview of the proteins associated with exposure to respirable dust and respirable crystalline silica (RCS) in construction workers in this study and gene ontology terms.

**Protein symbol (alternative names)**	**Protein name**	**Gene Ontology terms (biological process)**
Respirable dust		
DKK1	Dickkopf-related protein 1	Heart development
GH	Growth hormone	MAPK cascade
HO-1 (HMOX1)	Heme oxygenase 1	Angiogenesis, blood vessel morphogenesis, catabolic processes, immune response, regulation of blood pressure, response to hypoxia
PDGF subunit B	Platelet-derived growth factor subunit B	Coagulation, heart development, MAPK cascade, platelet activation, regulation of blood pressure, response to hypoxia
SCF	Stem cell factor	Cell adhesion, MAPK cascade
TF (CD142, F3)	Tissue factor	Angiogenesis, coagulation, inflammatory response, proteolysis
RCS		
CA5A	Carbonic anhydrase 5A, mitochondrial	One-carbon metabolic process
CEACAM8 (CD66b)	Carcinoembryonic antigenrelated cell adhesion molecule 8	Immune response
HAOX1	Hydroxyacid oxidase 1	Catabolic processes
LOX-1 (OLR1)	Lectin-like oxidized LDL receptor 1	Cell adhesion, inflammatory response, proteolysis
TF (CD142, F3)	Tissue factor	Angiogenesis, coagulation, inflammatory response, proteolysis
